# Supplementary material for: Foxp1 suppresses cortical angiogenesis and attenuates HIF-1alpha signaling to promote neural progenitor cell maintenance
Source: EMBO Rep. 2024 Apr 10;25(5):9. doi: 10.1038/s44319-024-00131-8 (PMC11094073; doi:10.1038/s44319-024-00131-8)
Supplement: Supplementary file 1 — Expanded View Table 1 [file 44319_2024_131_MOESM1_ESM.pdf]

Table EV1. Top 80 upregulated genes in Foxp1^{cKO} cortex at E12.5.

Gene Name (Mus musculus)	Fold change	P value	Description
Ddn	6.65539649	4.34E-09	dendrin [Source:MGI Symbol;Acc:MGI:108101]
Gm29683	5.55131856	0.00081083	predicted gene, 29683 [Source:MGI Symbol;Acc:MGI:5588842]
Gm15283	4.01183319	1.66E-15	predicted gene 15283 [Source:MGI Symbol;Acc:MGI:3705161]
F630040K05Rik	3.98810861	3.55E-52	RIKEN cDNA F630040K05 gene [Source:MGI Symbol;Acc:MGI:4437734]
Rasgef1a	3.27179303	0.00053465	RasGEF domain family, member 1A [Source:MGI Symbol;Acc:MGI:1917977]
CT010460.2	3.11608167	0.00193561	
Fam19a1	3.0666215	0.00070148	family with sequence similarity 19, member A1 [Source:MGI Symbol;Acc:MGI:2443695]
Esm1	2.829631	3.05E-11	endothelial cell-specific molecule 1 [Source:MGI Symbol;Acc:MGI:1918940]
Kcne3	2.57756205	3.81E-15	potassium voltage-gated channel, Isk-related subfamily, gene 3 [Source:MGI Symbol;Acc:MGI:1891124]
Mctp1	2.51954838	9.53E-06	multiple C2 domains, transmembrane 1 [Source:MGI Symbol;Acc:MGI:1926021]
Adm	2.50655921	1.12E-10	adrenomedullin [Source:MGI Symbol;Acc:MGI:108058]
Ciart	2.48310475	6.80E-09	circadian associated repressor of transcription [Source:MGI Symbol;Acc:MGI:2684975]
Egr3	2.39720897	0.00041827	early growth response 3 [Source:MGI Symbol;Acc:MGI:1306780]
Angpt2	2.37654774	8.90E-12	angiopoietin 2 [Source:MGI Symbol;Acc:MGI:1202890]
Cntnap1	2.30991797	0.00022566	contactin associated protein-like 1 [Source:MGI Symbol;Acc:MGI:1858201]
Caln1	2.29886844	0.00070511	calneuron 1 [Source:MGI Symbol;Acc:MGI:2155987]
Bnip3	2.22446793	4.69E-35	BCL2/adenovirus E1B interacting protein 3 [Source:MGI Symbol;Acc:MGI:109326]
Rps3a2	2.18806411	8.52E-07	ribosomal protein S3A2 [Source:MGI Symbol;Acc:MGI:3642853]
Serpine1	2.15823855	3.73E-06	serine (or cysteine) peptidase inhibitor, clade E, member 1 [Source:MGI Symbol;Acc:MGI:97608]
Slc16a3	2.1459402	1.88E-12	solute carrier family 16 (monocarboxylic acid transporters), member 3 [Source:MGI Symbol;Acc:MGI:1933438]
Hpcal4	2.07681771	0.00122888	hippocalcin-like 4 [Source:MGI Symbol;Acc:MGI:2157521]
Otof	2.07208059	0.00195399	otoferlin [Source:MGI Symbol;Acc:MGI:1891247]
Plppr4	2.02429672	7.38E-06	phospholipid phosphatase related 4 [Source:MGI Symbol;Acc:MGI:106530]
8430408G22Rik	1.98774221	7.16E-10	RIKEN cDNA 8430408G22 gene [Source:MGI Symbol;Acc:MGI:1918730]
Ackr2	1.90982731	2.87E-07	atypical chemokine receptor 2 [Source:MGI Symbol;Acc:MGI:1891697]
Vegfa	1.82627743	1.54E-36	vascular endothelial growth factor A [Source:MGI Symbol;Acc:MGI:103178]
P4ha2	1.79677903	0.00012164	procollagen-proline, 2-oxoglutarate 4-dioxygenase (proline 4-hydroxylase), alpha II polypeptide [Source:MGI Symbol;Acc:MGI:894286]

Igfbp3	1.79406457	1.44E-11	insulin-like growth factor binding protein 3 [Source:MGI Symbol;Acc:MGI:96438]
Grm5	1.79178696	1.11E-05	glutamate receptor, metabotropic 5 [Source:MGI Symbol;Acc:MGI:1351342]
Pfkp	1.78998445	4.81E-13	phosphofructokinase, platelet [Source:MGI Symbol;Acc:MGI:1891833]
Rgs4	1.78474484	3.20E-05	regulator of G-protein signaling 4 [Source:MGI Symbol;Acc:MGI:108409]
Pdk1	1.76402988	1.35E-17	pyruvate dehydrogenase kinase, isoenzyme 1 [Source:MGI Symbol;Acc:MGI:1926119]
Stc2	1.76397292	4.01E-05	stanniocalcin 2 [Source:MGI Symbol;Acc:MGI:1316731]
Ier3	1.76376615	1.49E-05	immediate early response 3 [Source:MGI Symbol;Acc:MGI:104814]
Coch	1.73031956	0.00067348	cochlin [Source:MGI Symbol;Acc:MGI:1278313]
A2m	1.70106531	1.59E-06	alpha-2-macroglobulin [Source:MGI Symbol;Acc:MGI:2449119]
Cacna1i	1.68718652	0.00097619	calcium channel, voltage-dependent, alpha 1I subunit [Source:MGI Symbol;Acc:MGI:2178051]
Sptbn4	1.68218143	7.86E-05	spectrin beta, non-erythrocytic 4 [Source:MGI Symbol;Acc:MGI:1890574]
Gpr158	1.66897042	0.00028633	G protein-coupled receptor 158 [Source:MGI Symbol;Acc:MGI:2441697]
Spock3	1.6566705	0.00053719	sparc/osteonectin, cwcv and kazal-like domains proteoglycan 3 [Source:MGI Symbol;Acc:MGI:1920152]
Gm996	1.65486028	3.27E-05	predicted gene 996 [Source:MGI Symbol;Acc:MGI:2685842]
Caly	1.65031118	0.00020828	calcyon neuron-specific vesicular protein [Source:MGI Symbol;Acc:MGI:1915816]
Faim2	1.63361551	1.74E-05	Fas apoptotic inhibitory molecule 2 [Source:MGI Symbol;Acc:MGI:1919643]
Plppr5	1.61519519	1.47E-06	phospholipid phosphatase related 5 [Source:MGI Symbol;Acc:MGI:1923019]
Lgi1	1.60916945	2.20E-05	leucine-rich repeat LGI family, member 1 [Source:MGI Symbol;Acc:MGI:1861691]
Ptprn	1.60844122	0.00102085	protein tyrosine phosphatase, receptor type, N [Source:MGI Symbol;Acc:MGI:102765]
Grin1	1.57363908	1.11E-05	glutamate receptor, ionotropic, NMDA1 (zeta 1) [Source:MGI Symbol;Acc:MGI:95819]
Tfap2d	1.57185471	4.32E-07	transcription factor AP-2, delta [Source:MGI Symbol;Acc:MGI:2153466]
Gipr	1.55868747	1.49E-06	gastric inhibitory polypeptide receptor [Source:MGI Symbol;Acc:MGI:1352753]
Gt(ROSA)26Sor	1.54378507	7.36E-16	gene trap ROSA 26, Philippe Soriano [Source:MGI Symbol;Acc:MGI:104735]
Ppp1r14c	1.53532665	2.29E-06	protein phosphatase 1, regulatory (inhibitor) subunit 14c [Source:MGI Symbol;Acc:MGI:1923392]
Pitpnm3	1.53304006	0.00089264	PITPNM family member 3 [Source:MGI Symbol;Acc:MGI:2685726]
Vldlr	1.5304937	4.82E-08	very low density lipoprotein receptor [Source:MGI Symbol;Acc:MGI:98935]
Ddit4	1.52883808	7.82E-07	DNA-damage-inducible transcript 4 [Source:MGI Symbol;Acc:MGI:1921997]
Grin2b	1.52478075	5.74E-05	glutamate receptor, ionotropic, NMDA2B (epsilon 2) [Source:MGI Symbol;Acc:MGI:95821]
Fstl4	1.52292707	0.00011112	follistatin-like 4 [Source:MGI Symbol;Acc:MGI:2443199]

Tnfaip3	1.52240613	9.99E-06	tumor necrosis factor, alpha-induced protein 3 [Source:MGI Symbol;Acc:MGI:1196377]
Flt1	1.52183643	3.06E-09	FMS-like tyrosine kinase 1 [Source:MGI Symbol;Acc:MGI:95558]
Arpp21	1.51578087	2.12E-06	cyclic AMP-regulated phosphoprotein, 21 [Source:MGI Symbol;Acc:MGI:107562]
Pfkfb3	1.50878807	3.17E-06	6-phosphofructo-2-kinase/fructose-2,6-biphosphatase 3 [Source:MGI Symbol;Acc:MGI:2181202]
Ndufa4l2	1.48736539	0.00014423	NADH dehydrogenase (ubiquinone) 1 alpha subcomplex, 4-like 2 [Source:MGI Symbol;Acc:MGI:3039567]
Ablim3	1.47532395	0.00069143	actin binding LIM protein family, member 3 [Source:MGI Symbol;Acc:MGI:2442582]
C1ql3	1.45828294	0.00160106	C1q-like 3 [Source:MGI Symbol;Acc:MGI:2387350]
Unc80	1.45537429	0.00113367	unc-80, NALCN activator [Source:MGI Symbol;Acc:MGI:2652882]
Pde8b	1.44773377	4.92E-06	phosphodiesterase 8B [Source:MGI Symbol;Acc:MGI:2443999]
Aldh1l1	1.4451944	0.00036134	aldehyde dehydrogenase 1 family, member L1 [Source:MGI Symbol;Acc:MGI:1340024]
Camk2b	1.43892756	7.04E-07	calcium/calmodulin-dependent protein kinase II, beta [Source:MGI Symbol;Acc:MGI:88257]
Dync1i1	1.43545093	1.85E-06	dynein cytoplasmic 1 intermediate chain 1 [Source:MGI Symbol;Acc:MGI:107743]
Lhfpl3	1.43425078	0.0008372	lipoma HMGIC fusion partner-like 3 [Source:MGI Symbol;Acc:MGI:1925076]
Rimbp2	1.43058043	0.00137117	RIMS binding protein 2 [Source:MGI Symbol;Acc:MGI:2443235]
Prelid2	1.4273582	5.63E-08	PRELI domain containing 2 [Source:MGI Symbol;Acc:MGI:1924869]
Scg2	1.42037754	1.98E-05	secretogranin II [Source:MGI Symbol;Acc:MGI:103033]
Ntsr1	1.41471679	0.00025169	neurotensin receptor 1 [Source:MGI Symbol;Acc:MGI:97386]
Eef1a2	1.40776743	6.59E-06	eukaryotic translation elongation factor 1 alpha 2 [Source:MGI Symbol;Acc:MGI:1096317]
Egln3	1.39567427	4.49E-05	egl-9 family hypoxia-inducible factor 3 [Source:MGI Symbol;Acc:MGI:1932288]
Ndrg1	1.3872471	0.00018288	N-myc downstream regulated gene 1 [Source:MGI Symbol;Acc:MGI:1341799]
Adap1	1.38600031	0.00100473	ArfGAP with dual PH domains 1 [Source:MGI Symbol;Acc:MGI:2442201]
Rgs6	1.38567624	0.00068082	regulator of G-protein signaling 6 [Source:MGI Symbol;Acc:MGI:1354730]
P4ha1	1.36681882	4.75E-09	procollagen-proline, 2-oxoglutarate 4-dioxygenase (proline 4-hydroxylase), alpha 1 polypeptide [Source:MGI Symbol;Acc:MGI:97463]
Adora2a	1.36165565	0.00130986	adenosine A2a receptor [Source:MGI Symbol;Acc:MGI:99402]
