# Supplementary material for: Foxp1 suppresses cortical angiogenesis and attenuates HIF-1alpha signaling to promote neural progenitor cell maintenance
Source: EMBO Rep. 2024 Apr 10;25(5):9. doi: 10.1038/s44319-024-00131-8 (PMC11094073; doi:10.1038/s44319-024-00131-8)
Supplement: Supplementary file 2 — Expanded View Table 2 [file 44319_2024_131_MOESM2_ESM.pdf]

Table EV2. Top 80 downregulated genes in Foxp1^{cKO} cortex at E12.5.

Gene Name (Mus musculus)	*Fold change*	*P value*	*Description*
Gm21887	-1.7454143	2.93E-05	predicted gene, 21887 [Source:MGI Symbol;Acc:MGI:5434051]
Txnip	-1.73318	3.03E-12	thioredoxin interacting protein [Source:MGI Symbol;Acc:MGI:1889549]
C030037D09Rik	-1.6809915	2.74E-05	RIKEN cDNA C030037D09 gene [Source:MGI Symbol;Acc:MGI:1924865]
AA465934	-1.6576161	4.33E-05	expressed sequence AA465934 [Source:MGI Symbol;Acc:MGI:2671018]
9930014A18Rik	-1.1108228	0.00056841	RIKEN cDNA 9930014A18 gene [Source:MGI Symbol;Acc:MGI:2444091]
Ccne1	-1.1039293	1.22E-05	cyclin E1 [Source:MGI Symbol;Acc:MGI:88316]
1600010M07Rik	-1.0650143	0.00184514	RIKEN cDNA 1600010M07 gene [Source:MGI Symbol;Acc:MGI:1917031]
Il31ra	-0.9216888	1.40E-14	interleukin 31 receptor A [Source:MGI Symbol;Acc:MGI:2180511]
Snapc5	-0.8809404	4.82E-08	small nuclear RNA activating complex, polypeptide 5 [Source:MGI Symbol;Acc:MGI:1914282]
Gm15564	-0.8262105	1.63E-06	predicted gene 15564 [Source:MGI Symbol;Acc:MGI:3783013]
Rpp25l	-0.8001076	0.0001774	ribonuclease P/MRP 25 subunit-like [Source:MGI Symbol;Acc:MGI:1917211]
Gm4430	-0.7812544	0.00072593	predicted gene 4430 [Source:MGI Symbol;Acc:MGI:3782614]
4930427A07Rik	-0.7579571	4.38E-05	RIKEN cDNA 4930427A07 gene [Source:MGI Symbol;Acc:MGI:2144738]
Dhrs4	-0.7578561	0.00076148	dehydrogenase/reductase (SDR family) member 4 [Source:MGI Symbol;Acc:MGI:90169]
Rfc4	-0.7527353	2.59E-05	replication factor C (activator 1) 4 [Source:MGI Symbol;Acc:MGI:2146571]
Mcur1	-0.7456268	0.00075977	mitochondrial calcium uniporter regulator 1 [Source:MGI Symbol;Acc:MGI:1923387]
Ung	-0.7436174	0.00051431	uracil DNA glycosylase [Source:MGI Symbol;Acc:MGI:109352]
Rps3a1	-0.7167743	0.00015373	ribosomal protein S3A1 [Source:MGI Symbol;Acc:MGI:1202063]
Slc25a10	-0.7167095	2.32E-05	solute carrier family 25 (mitochondrial carrier, dicarboxylate transporter), member 10 [Source:MGI Symbol;Acc:MGI:1353497]
Ide	-0.7047055	0.00040817	insulin degrading enzyme [Source:MGI Symbol;Acc:MGI:96412]
Rcc1	-0.6926344	0.00028344	regulator of chromosome condensation 1 [Source:MGI Symbol;Acc:MGI:1913989]
2310011J03Rik	-0.690648	0.00195057	RIKEN cDNA 2310011J03 gene [Source:MGI Symbol;Acc:MGI:1913624]
Prdx1	-0.6842499	0.00053835	peroxiredoxin 1 [Source:MGI Symbol;Acc:MGI:99523]
Myb	-0.6799152	0.00090893	myeloblastosis oncogene [Source:MGI Symbol;Acc:MGI:97249]
Pno1	-0.6761335	1.35E-05	partner of NOB1 homolog [Source:MGI Symbol;Acc:MGI:1913499]
Tyms	-0.6738263	8.02E-05	thymidylate synthase [Source:MGI Symbol;Acc:MGI:98878]

Arrdc4	-0.6635273	2.16E-05	arrestin domain containing 4 [Source:MGI Symbol;Acc:MGI:1913662]
Lsm5	-0.6616134	0.00048587	LSM5 homolog, U6 small nuclear RNA and mRNA degradation associated [Source:MGI Symbol;Acc:MGI:1913623]
Fam72a	-0.6612573	0.00018603	family with sequence similarity 72, member A [Source:MGI Symbol;Acc:MGI:1919669]
Amd1	-0.6491136	0.00060853	S-adenosylmethionine decarboxylase 1 [Source:MGI Symbol;Acc:MGI:88004]
Prx	-0.638529	0.00076685	periaxin [Source:MGI Symbol;Acc:MGI:108176]
Gcsh	-0.6369482	0.00137435	glycine cleavage system protein H (aminomethyl carrier) [Source:MGI Symbol;Acc:MGI:1915383]
Dut	-0.6356397	0.00034822	deoxyuridine triphosphatase [Source:MGI Symbol;Acc:MGI:1346051]
AC165092.1	-0.6350126	0.00127199	
Fanca	-0.6278163	0.0012161	Fanconi anemia, complementation group A [Source:MGI Symbol;Acc:MGI:1341823]
Pcna	-0.6278124	0.00050695	proliferating cell nuclear antigen [Source:MGI Symbol;Acc:MGI:97503]
Akr1b3	-0.6113962	0.00011939	aldo-keto reductase family 1, member B3 (aldose reductase) [Source:MGI Symbol;Acc:MGI:1353494]
Aurka	-0.6080558	0.00152722	aurora kinase A [Source:MGI Symbol;Acc:MGI:894678]
Prokr1	-0.6043059	0.00037827	prokineticin receptor 1 [Source:MGI Symbol;Acc:MGI:1929676]
Ccdc25	-0.5995813	3.90E-07	coiled-coil domain containing 25 [Source:MGI Symbol;Acc:MGI:1914429]
Pclaf	-0.5913014	2.02E-05	PCNA clamp associated factor [Source:MGI Symbol;Acc:MGI:1915276]
Timm22	-0.5798368	7.52E-05	translocase of inner mitochondrial membrane 22 [Source:MGI Symbol;Acc:MGI:1929742]
Cycs	-0.578209	1.86E-05	cytochrome c, somatic [Source:MGI Symbol;Acc:MGI:88578]
Dhfr	-0.5775575	0.00042771	dihydrofolate reductase [Source:MGI Symbol;Acc:MGI:94890]
Utp6	-0.5743641	0.00065969	UTP6 small subunit processome component [Source:MGI Symbol;Acc:MGI:2445193]
Mrpl12	-0.5672068	7.76E-05	mitochondrial ribosomal protein L12 [Source:MGI Symbol;Acc:MGI:1926273]
Tbl3	-0.5631961	0.00135835	transducin (beta)-like 3 [Source:MGI Symbol;Acc:MGI:2384863]
Umps	-0.5599798	0.00191753	uridine monophosphate synthetase [Source:MGI Symbol;Acc:MGI:1298388]
Larp7	-0.5587161	7.48E-06	La ribonucleoprotein domain family, member 7 [Source:MGI Symbol;Acc:MGI:107634]
Pole3	-0.5569267	0.00156599	polymerase (DNA directed), epsilon 3 (p17 subunit) [Source:MGI Symbol;Acc:MGI:1933378]
Ahcy	-0.5559686	2.52E-06	S-adenosylhomocysteine hydrolase [Source:MGI Symbol;Acc:MGI:87968]
Rrm2	-0.5538147	2.64E-05	ribonucleotide reductase M2 [Source:MGI Symbol;Acc:MGI:98181]
Wdhd1	-0.5485145	0.00115459	WD repeat and HMG-box DNA binding protein 1 [Source:MGI Symbol;Acc:MGI:2443514]
Slbp	-0.5479877	0.00032909	stem-loop binding protein [Source:MGI Symbol;Acc:MGI:108402]

Rfc3	-0.5449692	1.38E-06	replication factor C (activator 1) 3 [Source:MGI Symbol;Acc:MGI:1916513]
Cdc6	-0.5439973	0.00125598	cell division cycle 6 [Source:MGI Symbol;Acc:MGI:1345150]
Tubd1	-0.5430312	0.00131465	tubulin, delta 1 [Source:MGI Symbol;Acc:MGI:1891826]
Ppa1	-0.5374194	2.60E-08	pyrophosphatase (inorganic) 1 [Source:MGI Symbol;Acc:MGI:97831]
Spdl1	-0.5369133	5.73E-05	spindle apparatus coiled-coil protein 1 [Source:MGI Symbol;Acc:MGI:1917635]
Limd2	-0.5353452	0.00029489	LIM domain containing 2 [Source:MGI Symbol;Acc:MGI:1915053]
Rrm1	-0.5334689	0.00046298	ribonucleotide reductase M1 [Source:MGI Symbol;Acc:MGI:98180]
2310033P09Rik	-0.5333676	0.00183879	RIKEN cDNA 2310033P09 gene [Source:MGI Symbol;Acc:MGI:1915112]
Atp5k	-0.5296294	8.18E-05	ATP synthase, H+ transporting, mitochondrial F1F0 complex, subunit E [Source:MGI Symbol;Acc:MGI:106636]
Capza1	-0.5262706	0.00175806	capping protein (actin filament) muscle Z-line, alpha 1 [Source:MGI Symbol;Acc:MGI:106227]
Prim1	-0.5248936	0.00178259	DNA primase, p49 subunit [Source:MGI Symbol;Acc:MGI:97757]
Itpa	-0.5246876	0.00013457	inosine triphosphatase (nucleoside triphosphate pyrophosphatase) [Source:MGI Symbol;Acc:MGI:96622]
Psmc5	-0.5232555	0.00036823	protease (prosome, macropain) 26S subunit, ATPase 5 [Source:MGI Symbol;Acc:MGI:105047]
Hmgn2	-0.5217042	0.00024074	high mobility group nucleosomal binding domain 2 [Source:MGI Symbol;Acc:MGI:96136]
Phf5a	-0.5197565	0.00162192	PHD finger protein 5A [Source:MGI Symbol;Acc:MGI:2156864]
Nudcd2	-0.5191933	0.00146025	NudC domain containing 2 [Source:MGI Symbol;Acc:MGI:1277103]
Alyref	-0.5186022	1.03E-05	Aly/REF export factor [Source:MGI Symbol;Acc:MGI:1341044]
Tsfm	-0.5173147	0.00064066	Ts translation elongation factor, mitochondrial [Source:MGI Symbol;Acc:MGI:1913649]
Heatr1	-0.5163531	0.00069873	HEAT repeat containing 1 [Source:MGI Symbol;Acc:MGI:2442524]
Cenpu	-0.5144973	0.00123	centromere protein U [Source:MGI Symbol;Acc:MGI:1919126]
Dnajc9	-0.5127613	0.00166414	DnaJ heat shock protein family (Hsp40) member C9 [Source:MGI Symbol;Acc:MGI:1915326]
Arpc5l	-0.5114661	6.27E-05	actin related protein 2/3 complex, subunit 5-like [Source:MGI Symbol;Acc:MGI:1921442]
Pola1	-0.5094492	2.19E-06	polymerase (DNA directed), alpha 1 [Source:MGI Symbol;Acc:MGI:99660]
Ppil1	-0.5067604	9.25E-05	peptidylprolyl isomerase (cyclophilin)-like 1 [Source:MGI Symbol;Acc:MGI:1916066]
Abcf2	-0.5054141	0.00140308	ATP-binding cassette, sub-family F (GCN20), member 2 [Source:MGI Symbol;Acc:MGI:1351657]
Erh	-0.5036326	0.00135281	enhancer of rudimentary homolog (Drosophila) [Source:MGI Symbol;Acc:MGI:108089]
