# Supplementary material for: Foxp1 suppresses cortical angiogenesis and attenuates HIF-1alpha signaling to promote neural progenitor cell maintenance
Source: EMBO Rep. 2024 Apr 10;25(5):9. doi: 10.1038/s44319-024-00131-8 (PMC11094073; doi:10.1038/s44319-024-00131-8)
Supplement: Supplementary file 3 — Expanded View Table 3 [file 44319_2024_131_MOESM3_ESM.pdf]

Table EV3 Top 25 subcellular compartments associated with downregulated genes in Foxp1cKO cortex at E12.5.

Subcellular Localization (Compartment)	Strength
Cyclin E1-CDK2 complex	2.38
Alpha DNA polymerase:primase complex	2.26
DNA replication factor C complex	2.08
Ctf18 RFC-like complex	2.01
Cytosolic proteasome complex	1.9
DNA polymerase complex	1.76
Nuclear replication fork	1.71
Replisome	1.65
Replication fork	1.59
Cyclin-dependent protein kinase holoenzyme complex	1.44
Small nuclear ribonucleoprotein complex	1.25
DNA-directed RNA polymerase complex	1.19
Serine/threonine protein kinase complex	1.15
Transferase complex, transferring phosphorus-containing groups	1.02
Nuclear chromosome	0.87
Chromosome	0.73
Ribonucleoprotein complex	0.73
Transferase complex	0.63
Intracellular organelle lumen	0.6
Nuclear lumen	0.58
Mitochondrion	0.53
Catalytic complex	0.49
Intracellular non-membrane-bounded organelle	0.45
Cytosol	0.45
Nucleus	0.43
