# Supplementary material for: Foxp1 suppresses cortical angiogenesis and attenuates HIF-1alpha signaling to promote neural progenitor cell maintenance
Source: EMBO Rep. 2024 Apr 10;25(5):9. doi: 10.1038/s44319-024-00131-8 (PMC11094073; doi:10.1038/s44319-024-00131-8)
Supplement: Supplementary file 4 — Expanded View Table 4 [file 44319_2024_131_MOESM4_ESM.pdf]

Table EV4 Top 25 subcellular compartments associated with upregulated genes in Foxp1cKO cortex at E12.5.

6-phosphofructokinase complex	2.02
Procollagen-proline 4-dioxygenase complex	2.02
Dendritic branch	1.8
NMDA selective glutamate receptor complex	1.6
Glucose transporter complex	1.6
Growth factor complex	1.32
Synaptic cleft	1.3
Hippocampal mossy fiber to CA3 synapse	1.27
Calyx of Held	1.25
Parallel fiber to Purkinje cell synapse	1.2
Integral component of postsynaptic density membrane	1.13
Photoreceptor inner segment	1.13
Excitatory synapse	1.1
Postsynaptic density membrane	1.08
Main axon	1.07
Presynaptic active zone	1.05
Voltage-gated potassium channel complex	1.02
Integral component of postsynaptic membrane	0.98
Intrinsic component of synaptic membrane	0.95
Integral component of synaptic membrane	0.95
Presynaptic membrane	0.95
Intrinsic component of presynaptic membrane	0.95
Integral component of presynaptic membrane	0.92
Neuron to neuron synapse	0.9
Postsynaptic density	0.89
