# Supplementary material for: Foxp1 suppresses cortical angiogenesis and attenuates HIF-1alpha signaling to promote neural progenitor cell maintenance
Source: EMBO Rep. 2024 Apr 10;25(5):9. doi: 10.1038/s44319-024-00131-8 (PMC11094073; doi:10.1038/s44319-024-00131-8)
Supplement: Supplementary file 5 — Appendix [file 44319_2024_131_MOESM5_ESM.pdf]

Appendix.

Table of Contents.

Appendix Figure S1……………………………………………………………………page 2

Appendix Figure S2……………………………………………………………………page 3

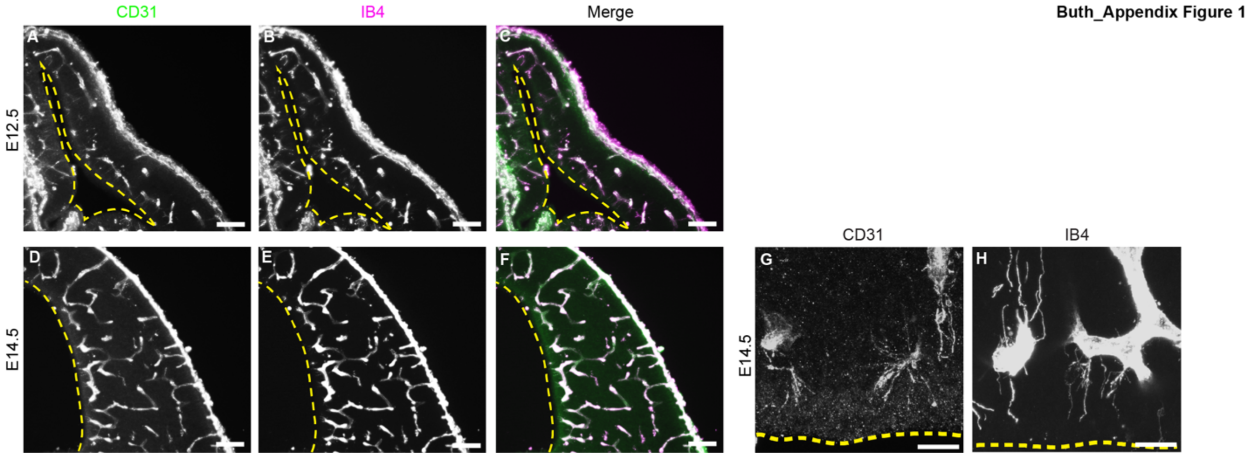

Appendix Figure S1 CD31 and Isolectin B4 staining in the cortical vasculature.

(A-C) Immunohistochemistry for CD31 and Isolectin B4 in the wildtype cortex at E12.5. (D-E) Immunohistochemistry for CD31 and Isolectin B4 in the wildtype cortex at E14.5. (G-H) High magnification images of filopodia labeled with CD31 and Isolectin B4 at the ventricular surface. Scale bars 100µm (A-F), 5 µm (G-H).

Buth_Appendix Figure 2

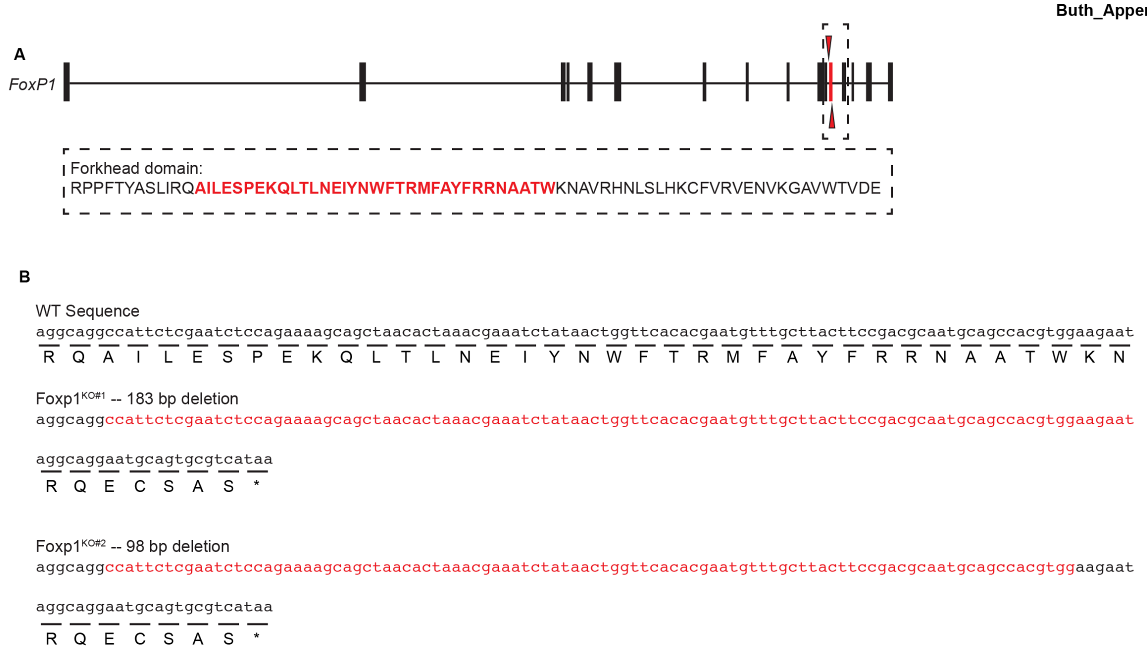

Appendix Figure S2 Design and Sequencing of Foxp1^{KO} mouse embryonic stem cell lines.

(A) Schematic of the mouse Foxp1 gene. Boxed area denotes the forkhead domain, exon in red is the exon targeted for excision. The boxed area below is the protein sequence of the forkhead domain, and the red text represents the predicted region to be removed. (B) Sequencing results from wildtype, Foxp1$^{KO\#1,}$ and Foxp1$^{KO\#}$2 mouse embryonic stem cell lines. The red text represents the region excised.
