# Supplementary figures and images for: Foxp1 suppresses cortical angiogenesis and attenuates HIF-1alpha signaling to promote neural progenitor cell maintenance
Source: EMBO Rep. 2024 Apr 10;25(5):9. doi: 10.1038/s44319-024-00131-8 (PMC11094073; doi:10.1038/s44319-024-00131-8)

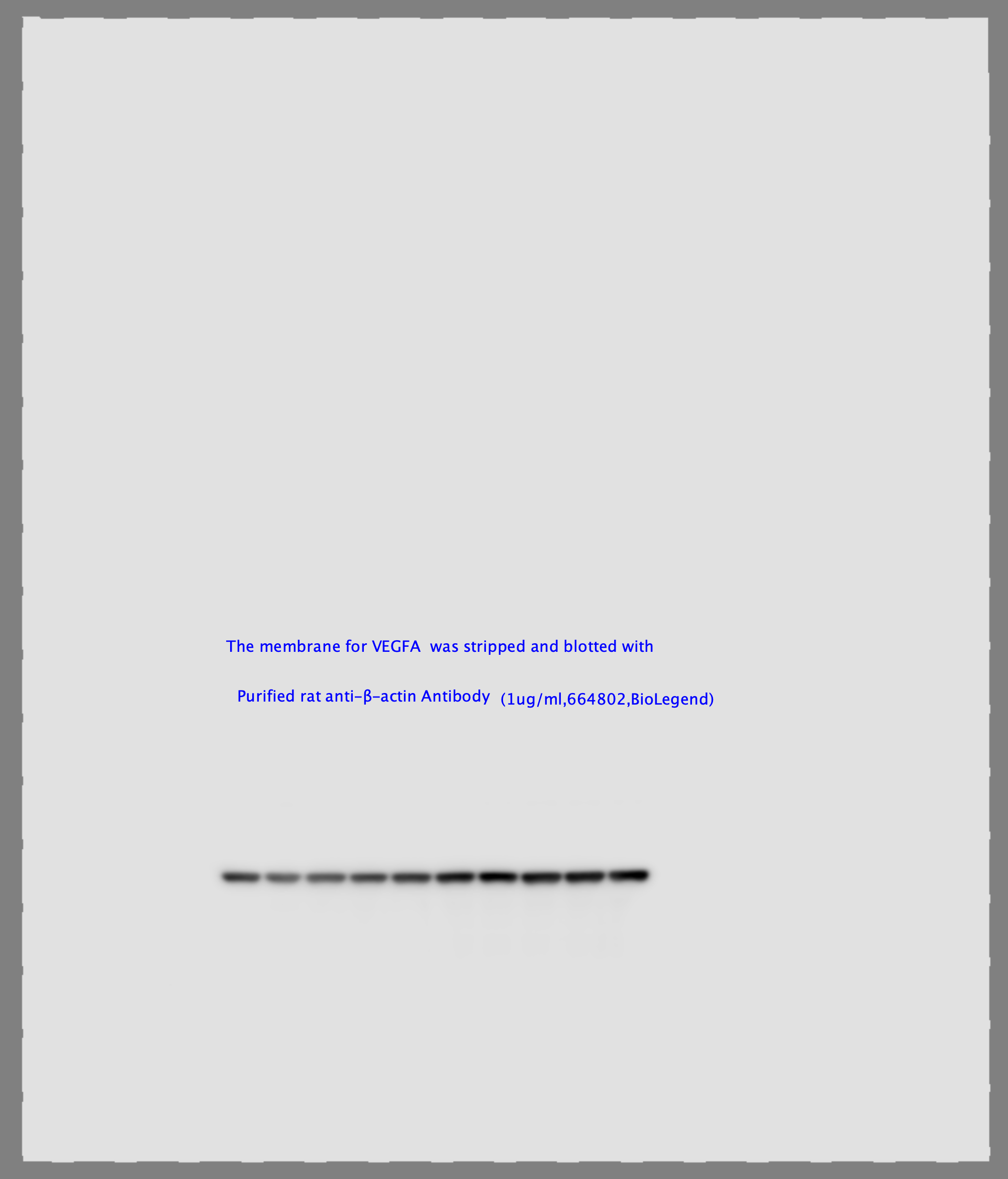

Supplement: Supplementary file 10 — Figure EV2T-X Source Data [file 44319_2024_131_MOESM10_ESM.zip › Western blots for Figure EV2/EV2T/EV2 VEGFa actin.tif]

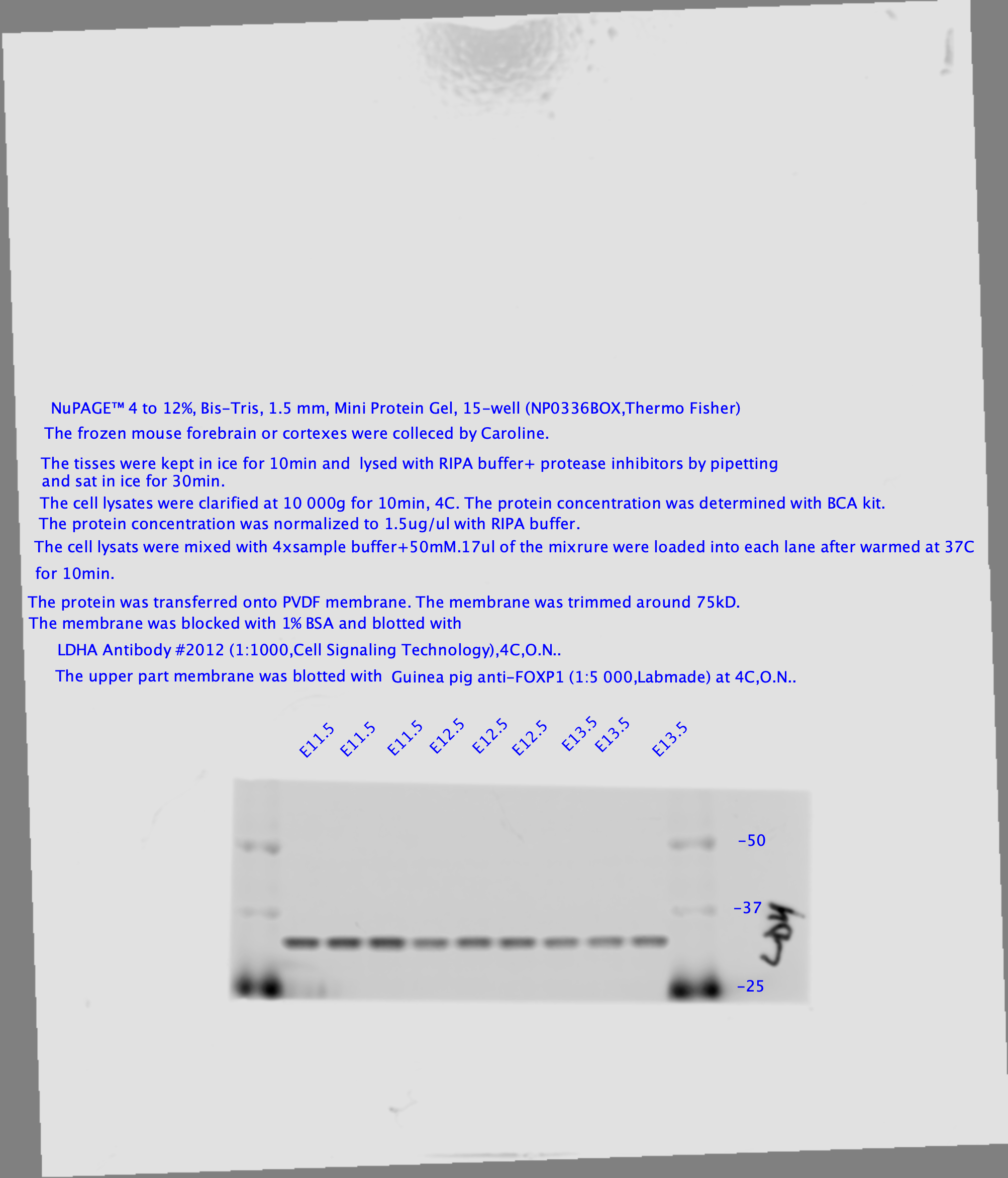

Supplement: Supplementary file 10 — Figure EV2T-X Source Data [file 44319_2024_131_MOESM10_ESM.zip › Western blots for Figure EV2/EV2T/EV2 Ldha.tif]

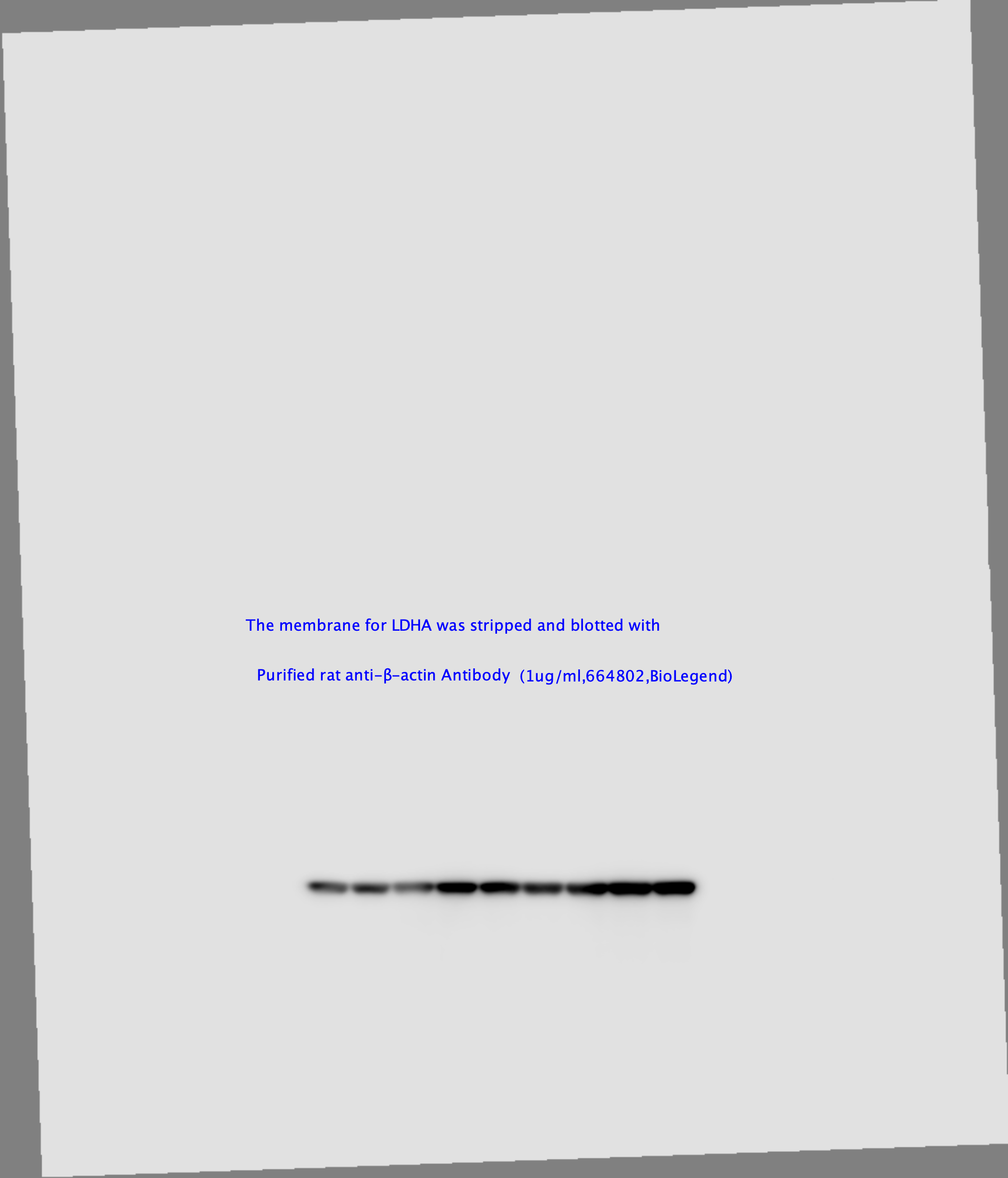

Supplement: Supplementary file 10 — Figure EV2T-X Source Data [file 44319_2024_131_MOESM10_ESM.zip › Western blots for Figure EV2/EV2T/EV2 LDHA actin.tif]

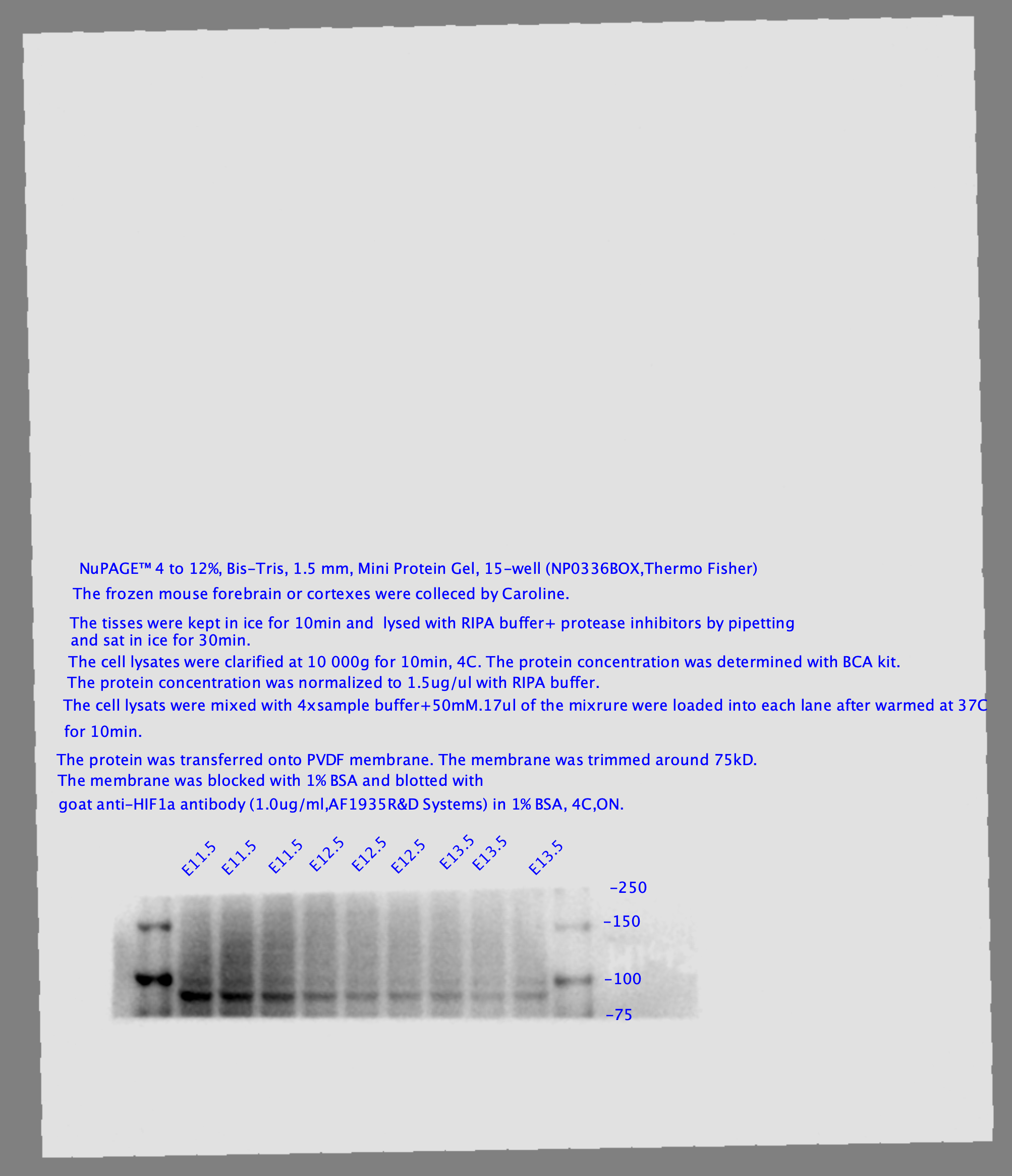

Supplement: Supplementary file 10 — Figure EV2T-X Source Data [file 44319_2024_131_MOESM10_ESM.zip › Western blots for Figure EV2/EV2T/EV2 HIF1a.tif]

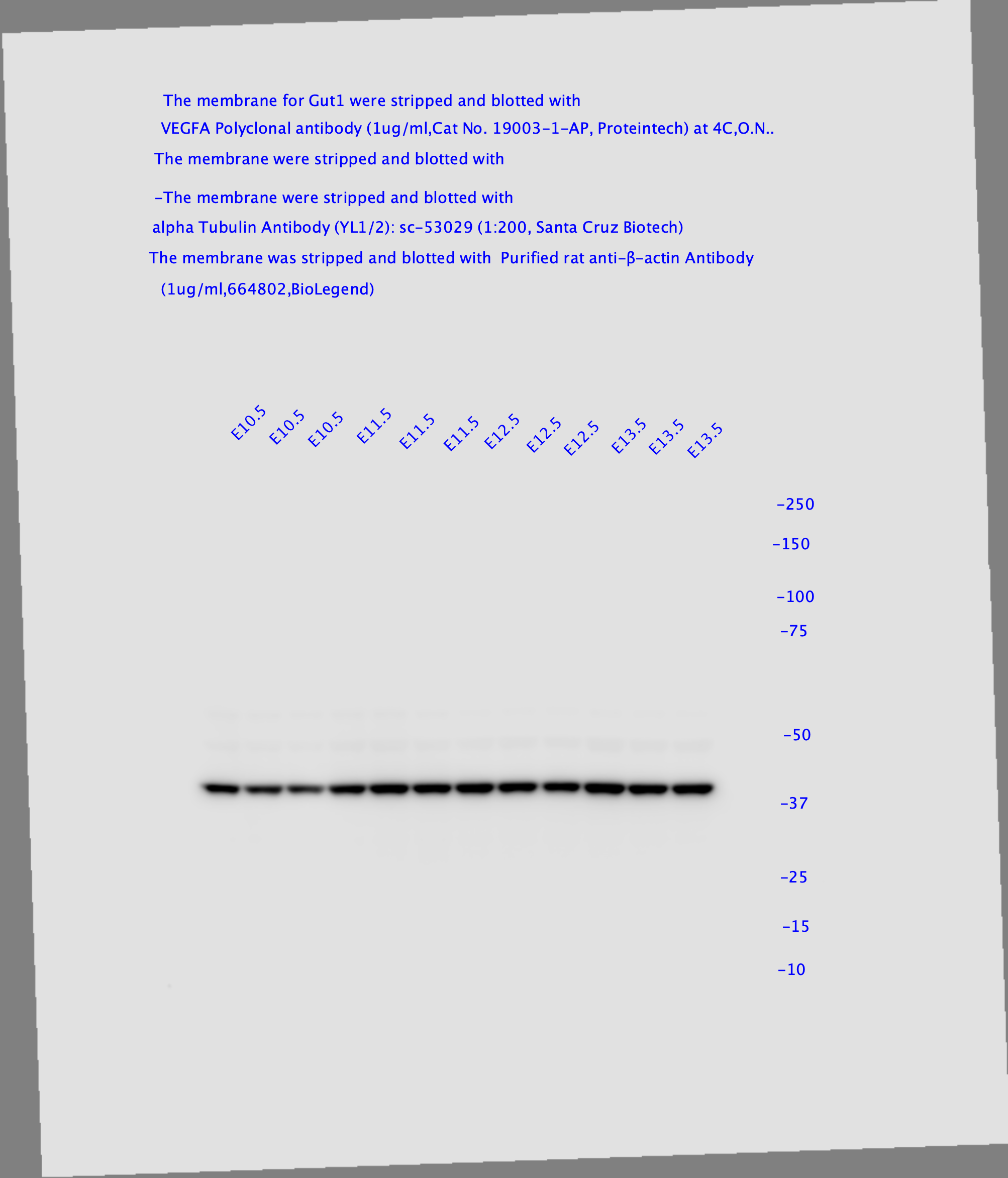

Supplement: Supplementary file 10 — Figure EV2T-X Source Data [file 44319_2024_131_MOESM10_ESM.zip › Western blots for Figure EV2/EV2T/EV2 Glut1 actin.tif]

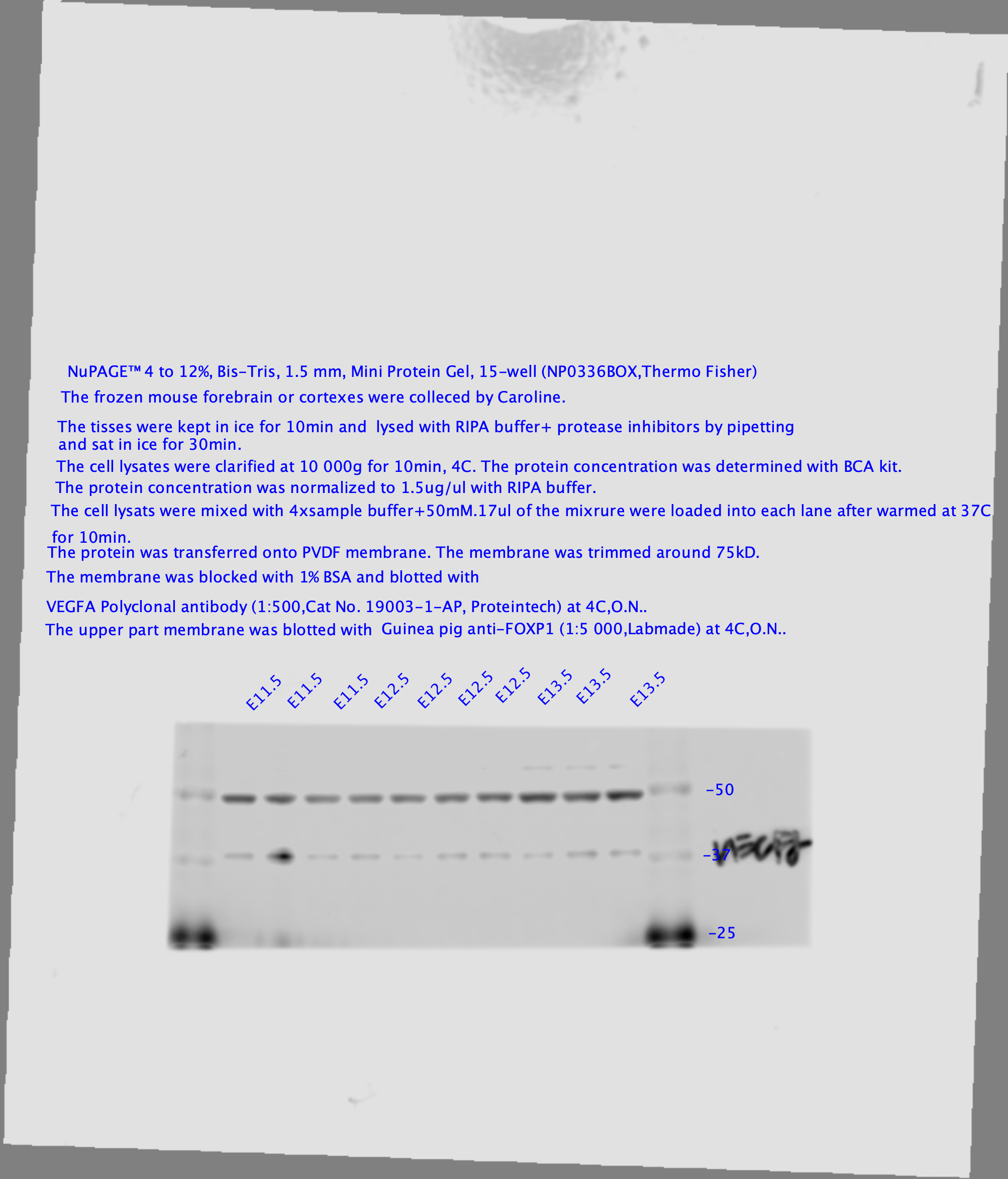

Supplement: Supplementary file 10 — Figure EV2T-X Source Data [file 44319_2024_131_MOESM10_ESM.zip › Western blots for Figure EV2/EV2T/EV2 VEGFa.tif]

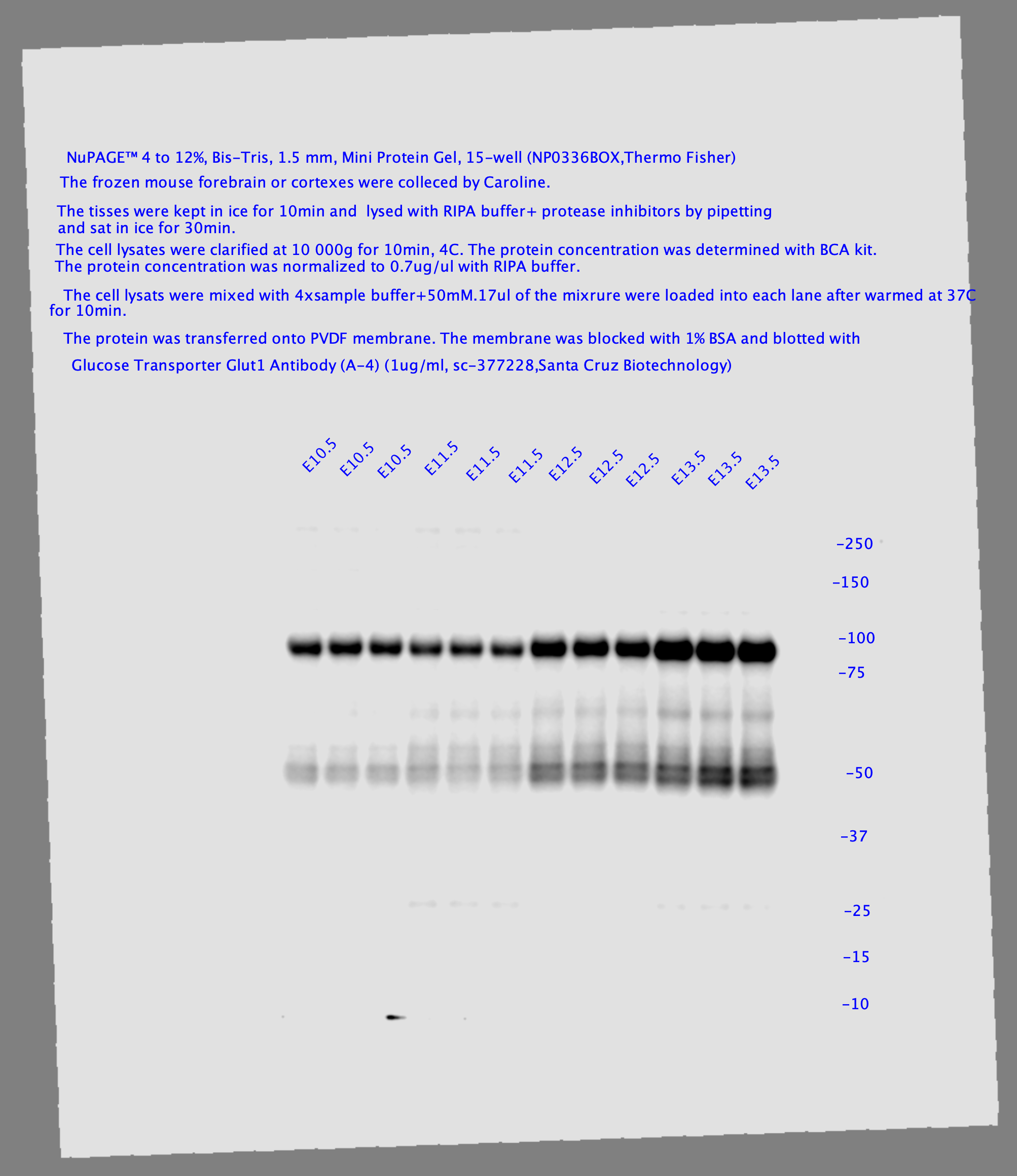

Supplement: Supplementary file 10 — Figure EV2T-X Source Data [file 44319_2024_131_MOESM10_ESM.zip › Western blots for Figure EV2/EV2T/EV2 Glut1.tif]

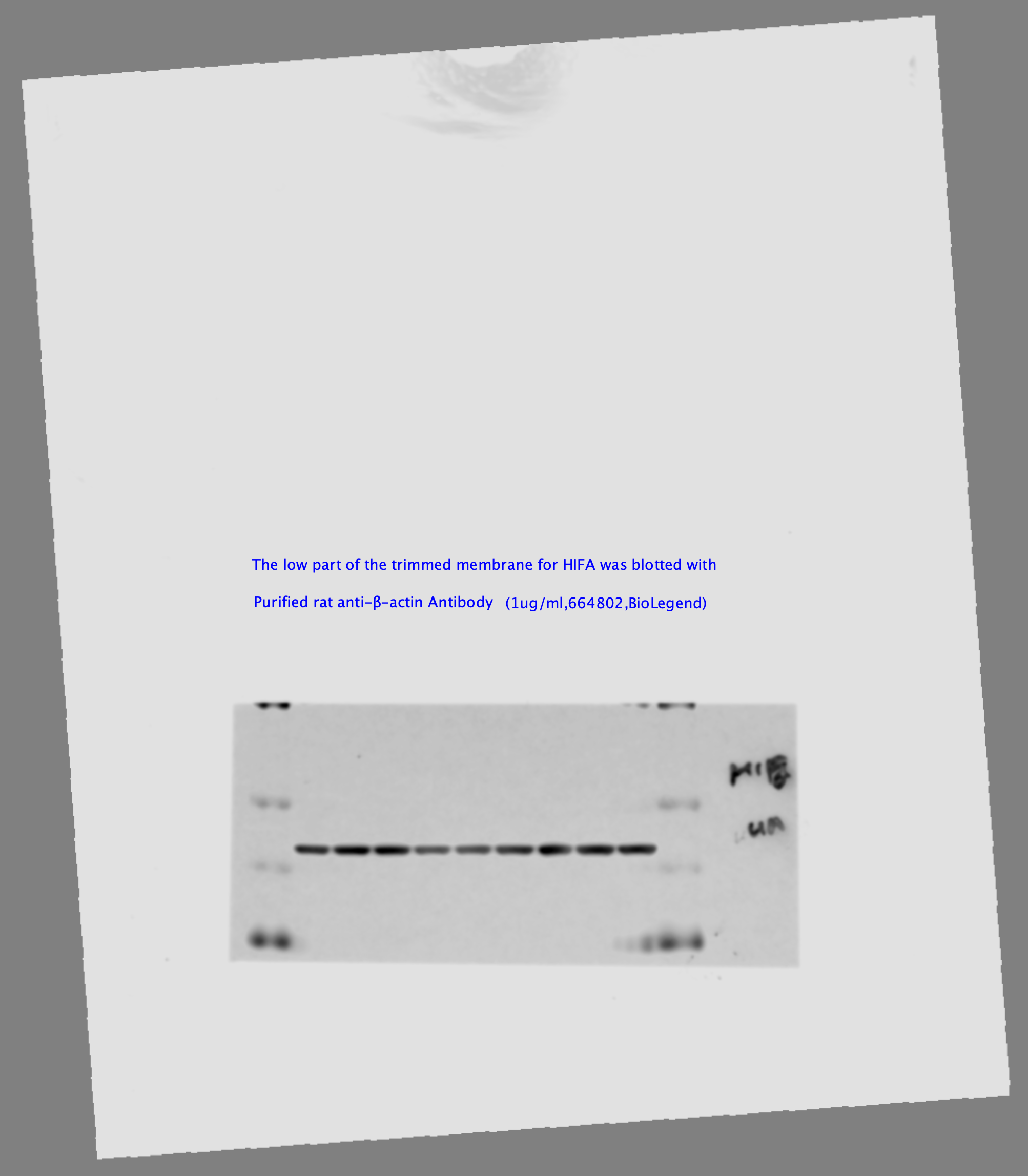

Supplement: Supplementary file 10 — Figure EV2T-X Source Data [file 44319_2024_131_MOESM10_ESM.zip › Western blots for Figure EV2/EV2T/EV2 HIF1a actin.tif]
