# Supplementary material for: Foxp1 suppresses cortical angiogenesis and attenuates HIF-1alpha signaling to promote neural progenitor cell maintenance
Source: EMBO Rep. 2024 Apr 10;25(5):9. doi: 10.1038/s44319-024-00131-8 (PMC11094073; doi:10.1038/s44319-024-00131-8)
Supplement: Supplementary file 12 — Expanded View Figures [file 44319_2024_131_MOESM12_ESM.pdf]

Expanded View Figures

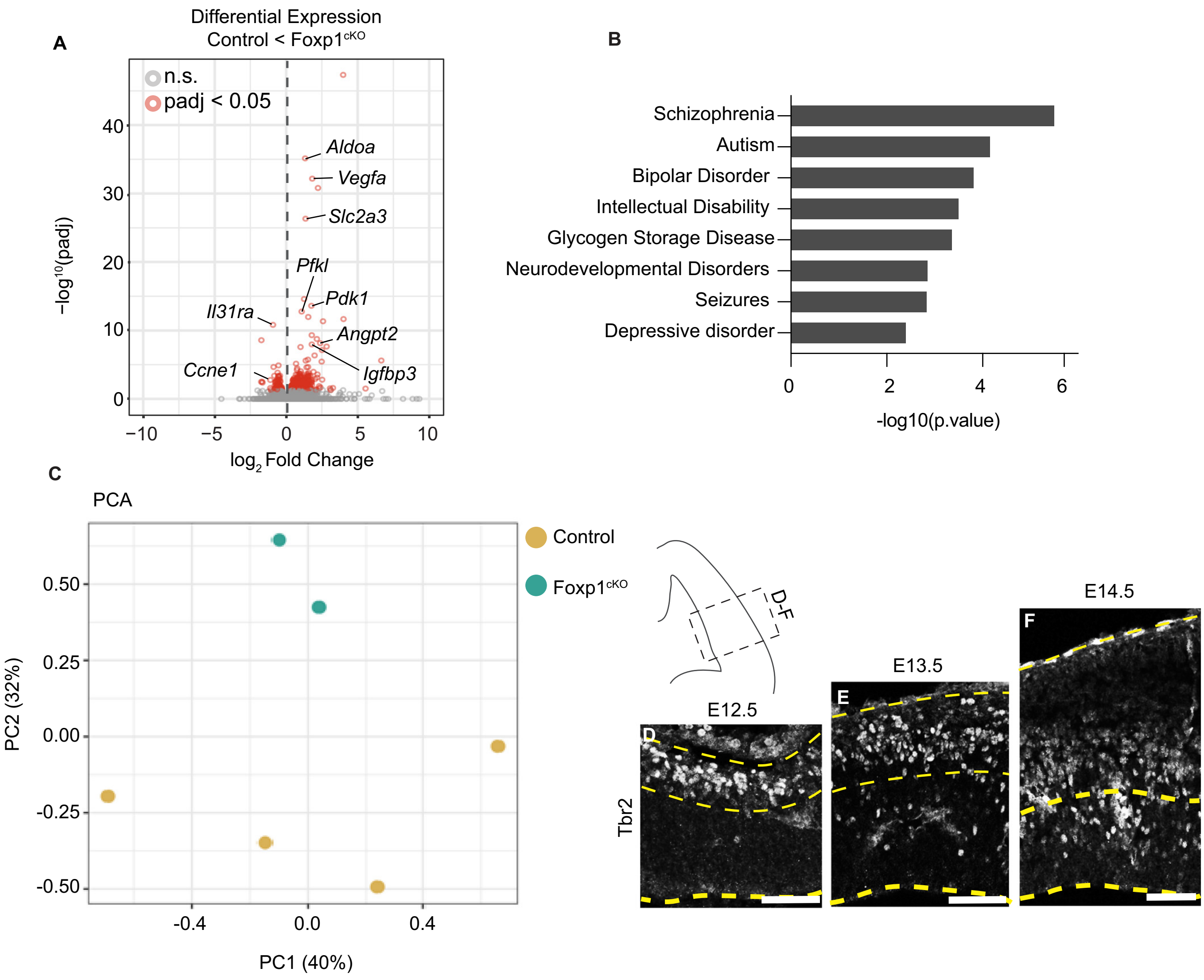

Figure EV1. RNA Seq analysis of Foxp1^{cKO} cortex.

(**A**) Volcano plot of gene expression changes in the absence of Foxp1 in E12.5 lateral cortex compared to control embryos. Gray circles denote non-significant gene changes (adjusted *p*-value > 0.05); red circles denote significantly differentially expressed genes (adjusted *p*-value < 0.05). (**B**) Human disorders associated with genes significantly misregulated in Foxp1^{cKO} mutants at E12.5. (**C**) The principal component analysis (PCA) of control and Foxp1cKO mutants shows PC1 and PC2. (**D–F**) IHC for Tbr2^{+} intermediate progenitors in wild-type cortex at E12.5, E13.5, and E14.5. Schematic denotes area image in (**D–F**). Scale bars 50 μm. Data information: significance determined by ANOVA (**A**, **B**).

 © The Author(s)

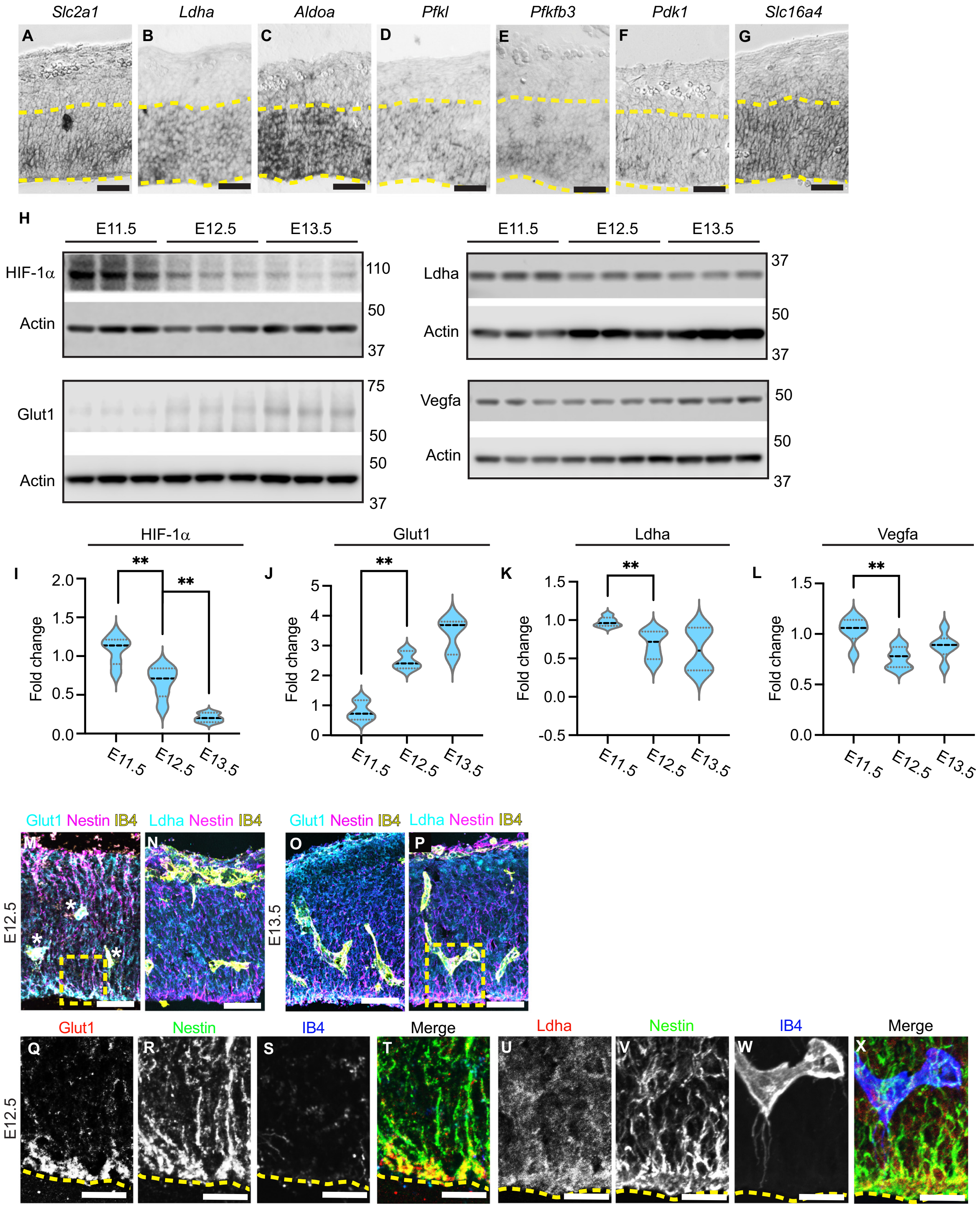
Slc2a1
Ldha
Aldoa
Pfkl
Pfkfb3
Pdk1
Slc16a4
A B C D E F G H
E11.5 E12.5 E13.5
HIF-1α
Actin
Glut1
Ldha
Vegfa
110 50 37 75 50
I J K L
Fold change
E11.5 E12.5 E13.5
**
Glut1 Nestin IB4
Ldha Nestin IB4
M N O P
E12.5
E13.5
Q R S T U V W X
Glut1
Nestin
IB4
Merge
Ldha

Figure EV2. HIF-1α target gene expression in RG in the wild-type cortex.

(**A–G**) Wild-type mRNA expression of glycolysis genes *Slc2a1, Ldha, Aldoa, Pfkl, Pfkfb3, Pdk1*, and *Slc16a4* in the wild-type lateral cortex at E12.5. (**H**) Western blot analysis of HIF-1α, Glut1, Ldha, and Vegfa (with Beta Actin) in wild-type cortical lysates at E11.5, E12.5, E13.5. (**I**) Fold change of HIF-1α levels normalized to Beta Actin between E11.5 and E13.5. (**J**) Fold change of Glut1 levels normalized to Beta Actin between E11.5 and E13.5. (**K**) Fold change of Ldha levels normalized to Beta Actin between E11.5 and E13.5. (**L**) Fold change of Vegfa levels normalized to Actin between E11.5 and E13.5. (**M**, **N**) IHC for Glut1 and Ldha with Isolectin B4, and Nestin at E12.5 in the wild-type cortex. (**O**, **P**) IHC for Glut1 or Ldha with Isolectin B4, and Nestin at E13.5 in the wild-type cortex. Boxed areas are magnified in (**Q–X**). (**Q–T**) High magnification image of IHC for Glut1 in Nestin+ RG at E12.5. Isolectin B4 labels blood vessels. (**U–X**) High magnification images of IHC for Ldha in Nestin+ RG at E13.5. Isolectin B4 labels blood vessels. Scale bars 50 μm (**A–K**), 10 μm (**L–S**). Data information: $N = 5$–9 embryos per time point, 3–6 replicates. $p = 0.0039$ and 0.0073 (**I**). $N = 5$–9 embryos per time point, 3 replicates. $p = 0.0028$ (**J**). $N = 5$–9 embryos per time point, 6 replicates. $p = 0.0041$ (**K**). $N = 5$–9 embryos per time point, 6 replicates. $p = 0.0017$ (**L**). All Student's t-tests. All data represented as mean ± SEM. Source data are available online for this figure.

 © The Author(s)

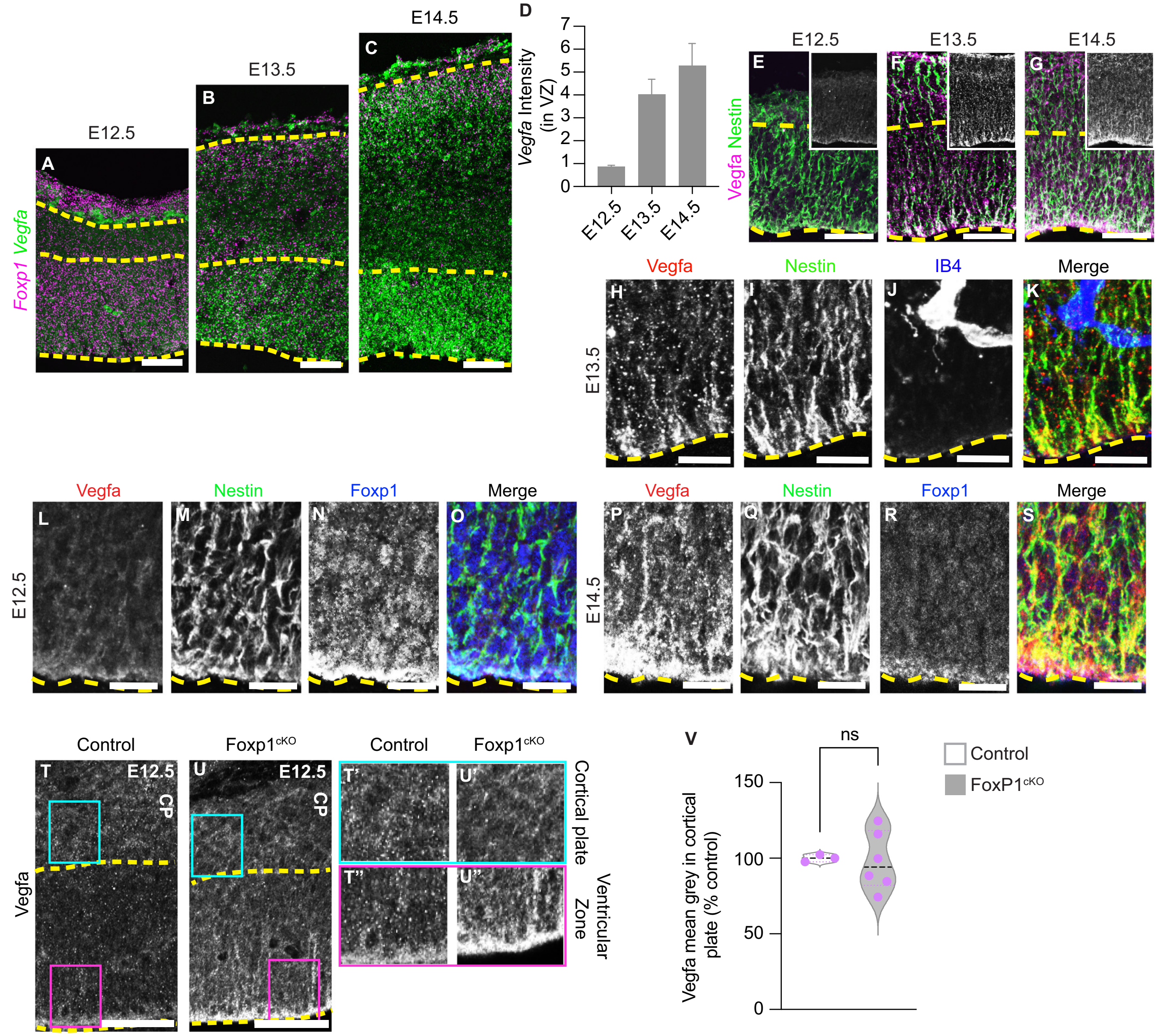

Figure EV3. Spatial and temporal relationship between Foxp1 and Vegfa in radial glia and neurons.

(**A–C**) RNA Scope analysis of Vegfa and Foxp1 in the wild-type cortex at E12.5–E14.5. (**D**) *Vegfa* mean gray value (over background) in the ventricular zone between E12.5–E14.5. (**E–G**) IHC for Vegfa and Nestin in the wild-type cortex at E12.5, E13.5, and E14.5. (**H–K**) High magnification images of Vegfa, Nestin, and IB4 at the apical surface of the VZ at E13.5. (**L–S**) High magnification images of IHC for Vegfa, Nestin, and Foxp1 at E12.5 and E14.5 in wild-type cortex. (**T–U**) IHC for Vegfa in the cortex at E12.5 in control and Foxp1^{cKO} embryos. Cyan boxes represent areas in CP magnified in **T**,′ and **U**.′ Magenta boxes represent the area within VZ magnified in **T**″ and **U**″. (**V**) Quantification of Vegfa mean gray values in the cortical plate in control and Foxp1^{cKO} cortex at E12.5. Scale bars 50 μm (**A–G**, **T**, **U**) 10 μm (**H–S**). Data information: $N = 5$–7 embryos per time point (**D**). $N = 3$ control, 6 mutant (2 litters). $p = 0.8674$, Student's t-test (**V**). All data represented as mean ± SEM.

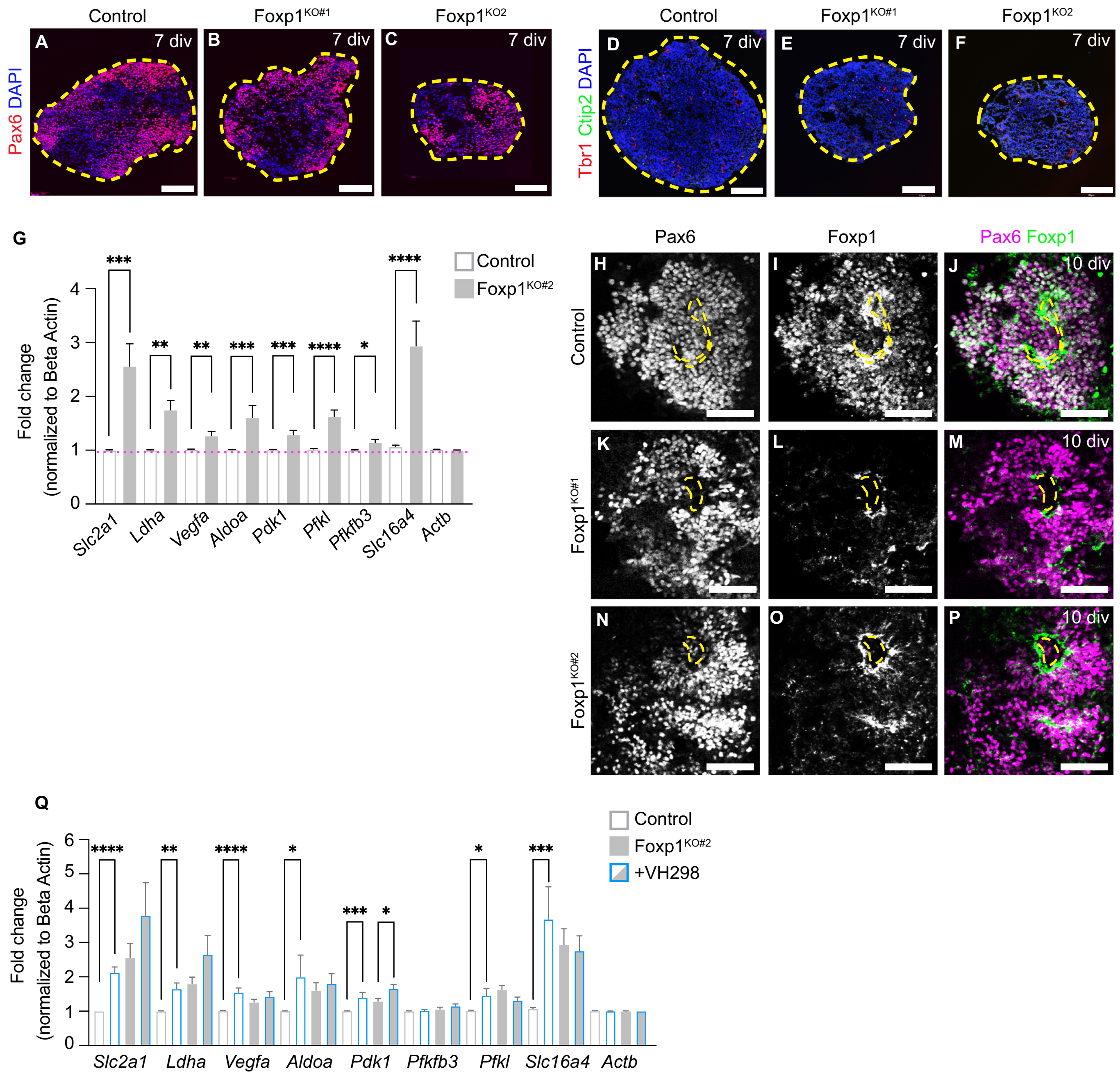

Figure EV4. Analysis of Foxp1-deficient cortical spheroids.

(**A–C**) IHC for Pax6 in control and Foxp1^{KO} spheroids at 7 days in vitro (div). (**D–F**) IHC for Ctip2 and Tbr1^{+} neurons in control and Foxp1^{KO} spheroids at 7 div. (**G**) qPCR analysis of HIF-1α target gene expression in control and Foxp1$^{KO\#2}$ spheroids at 10 div. (**H–P**) IHC for Foxp1 in Pax6^{+} NPCs in control and Foxp1^{KO} spheroids at 10 div. (**Q**) qPCR analysis of HIF-1α target gene expression in control and Foxp1$^{KO\#2}$ spheroids at 10 div. treated with DMSO or VH298. Scale bars 100 μm (**A–F**), 50 μm (**H–P**). Data information: $N = 10$–12 spheroids from 3 individual batches. $p = 0.0007$ (*Slc2a1*), 0.0108 (*Ldha*), 0.0031 (*Vegfa*), 0.0009 (*Aldoa*), 0.0002 (*Pdk1*), 0.0002 (*Pfkl*), 0.0226 (*Pfkfb3*), <0.0001 (*Slc16a4*). Student's t-test (**G**). $p =$ <0.0001 (*Slc2a1*, control), 0.0013 (*Ldha*, control) <0.0001 (*Vegfa*, control), 0.0179 (*Aldoa*, control) 0.0002 (*Pdk1*, control), 0.0312 (*Pdk1*, Foxp1$^{KO\#2}$), 0.0201 (*Pfkl*, control), 0.0003 (*Slc16a4*, control). Student's t-test (**Q**). All data represented as mean ± SEM.

 © The Author(s)
