# Supplementary material for: Foxp1 suppresses cortical angiogenesis and attenuates HIF-1alpha signaling to promote neural progenitor cell maintenance
Source: EMBO Rep. 2024 Apr 10;25(5):9. doi: 10.1038/s44319-024-00131-8 (PMC11094073; doi:10.1038/s44319-024-00131-8)
Supplement: Supplementary file 9 — Source data Fig. 4 [file 44319_2024_131_MOESM9_ESM.zip › Figure 4 zip files/4A.zip!/4A/4A.pdf]

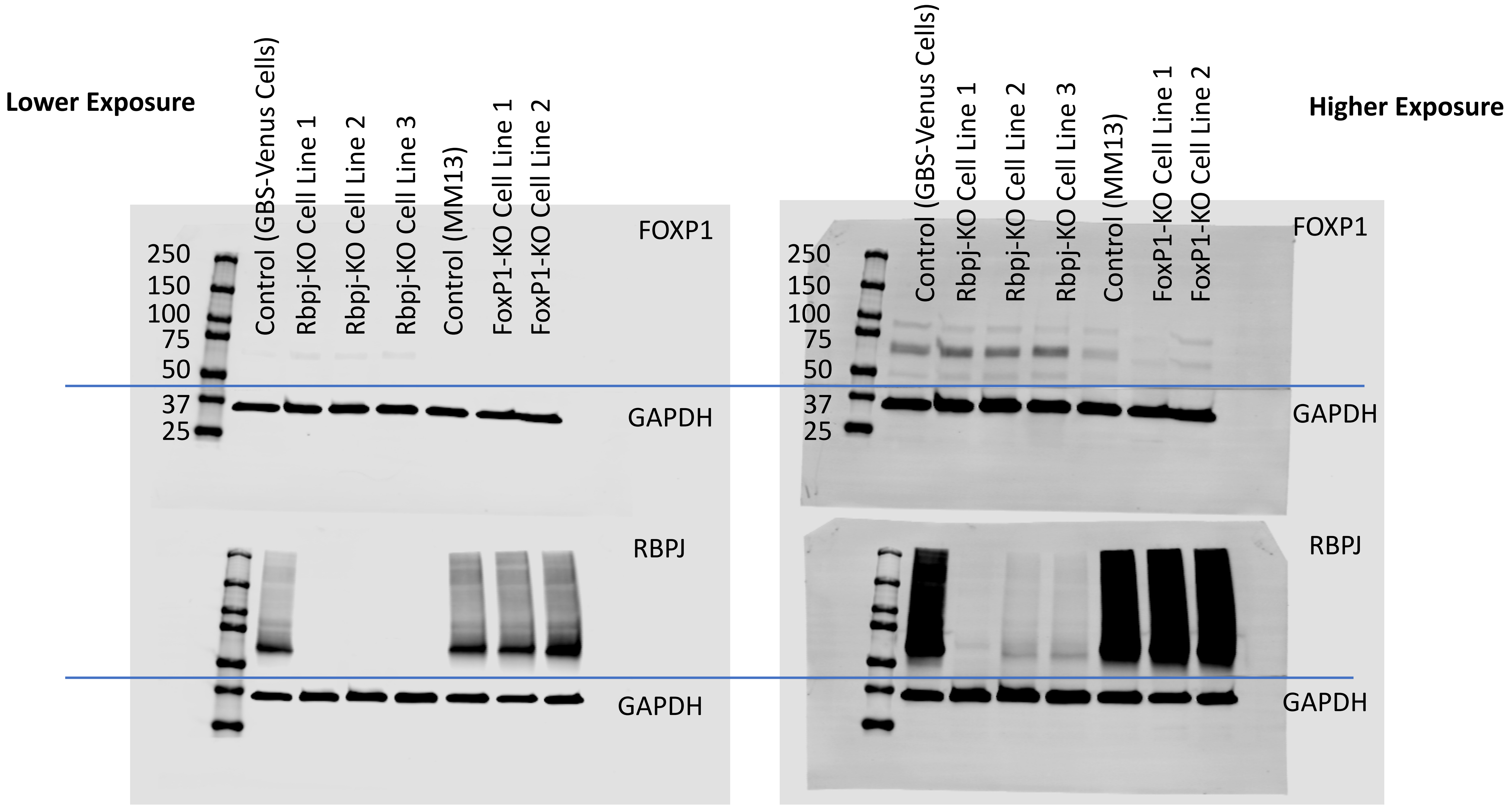

Lower Exposure
Control (GBS-Venus Cells)
Rbpj-KO Cell Line 1
Rbpj-KO Cell Line 2
Rbpj-KO Cell Line 3
Control (MM13)
FoxP1-KO Cell Line 1
FoxP1-KO Cell Line 2
FOXP1
250
150
100
75
50
37
25
GAPDH
RBPJ
GAPDH
Higher Exposure
Control (GBS-Venus Cells)
Rbpj-KO Cell Line 1
Rbpj-KO Cell Line 2
Rbpj-KO Cell Line 3
Control (MM13)
FoxP1-KO Cell Line 1
FoxP1-KO Cell Line 2
FOXP1
250
150
100
75
50
37
25
GAPDH
RBPJ
GAPDH
